# 0.75% ropivacaine may be a suitable drug in pregnant women undergoing urgent cesarean delivery during labor analgesia period

**DOI:** 10.1186/s12871-024-02597-4

**Published:** 2024-06-25

**Authors:** Xin Men, Qian Wang, Jia-fu Dong, Pei Chen, Xiao-xiao Qiu, Yin-qiu Han, Wei-long Wang, Jin Zhou, Hong-yan Shou, Zhen-feng Zhou

**Affiliations:** 1 Department of Anesthesiology, Hangzhou Women’s Hospital (Hangzhou Maternity and Child Health Care Hospital, Hangzhou First People’s Hospital Qianjiang New City Campus, Zhejiang Chinese Medical University), Hangzhou, 310008 China; 2 Department of Anesthesiology, Zhejiang Hospital, The Affiliated Zhejiang Hospital, Zhejiang University School of Medicine, Hangzhou, 310013 China

**Keywords:** Ropivacaine, Chloroprocaine, Urgent cesarean delivery, Epidural

## Abstract

**Background:**

3% chloroprocaine (CP) has been reported as the common local anesthetic used in pregnant women undergoing urgent cesarean delivery during labor analgesia period. However, 0.75% ropivacaine is considered a promising and effective alternative. Therefore, we conducted a randomized controlled trial to compare the effectiveness and safety of 0.75% ropivacaine with 3% chloroprocaine for extended epidural anesthesia in pregnant women.

**Methods:**

We conducted a double-blind, randomized, controlled, single-center study from November 1, 2022, to April 30, 2023. We selected forty-five pregnant women undergoing urgent cesarean delivery during labor analgesia period and randomized them to receive either 0.75% ropivacaine or 3% chloroprocaine in a 1:1 ratio. The primary outcome was the time to loss of cold sensation at the T4 level.

**Results:**

There was a significant difference between the two groups in the time to achieve loss of cold sensation (303, 95%CI 255 to 402 S vs. 372, 95%CI 297 to 630 S, *p* = 0.024). There was no significant difference the degree of motor block (*p* = 0.185) at the Th4 level. Fewer pregnant women required additional local anesthetics in the ropivacaine group compared to the chloroprocaine group (4.5% VS. 34.8%, *p* = 0.011). The ropivacaine group had lower intraoperative VAS scores (*p* = 0.023) and higher patient satisfaction scores (*p* = 0.040) than the chloroprocaine group. The incidence of intraoperative complications was similar between the two groups, and no serious complications were observed.

**Conclusions:**

Our study found that 0.75% ropivacaine was associated with less intraoperative pain treatment, higher patient satisfaction and reduced the onset time compared to 3% chloroprocaine in pregnant women undergoing urgent cesarean delivery during labor analgesia period. Therefore, 0.75% ropivacaine may be a suitable drug in pregnant women undergoing urgent cesarean delivery during labor analgesia period.

**Clinical trial number and registry URL:**

The registration number: ChiCTR2200065201; http://www.chictr.org.cn, Principal investigator: MEN, Date of registration: 31/10/2022.

## Introduction

In the United States, approximately 71% of women use epidural labor analgesia during labor [1]. Pain during vaginal delivery can lead to emotional distress, with deleterious effects on both the mother and baby. Epidural labor analgesia with appropriate analgesic effect reduces the use of prolatin, promotes the amount and duration of lactation, with a shorter onset of lactation and a lower incidence of delayed lactation [2]. Epidural analgesia does not significantly influence the newborn’s well-being during labour and delivery [3]. However the drug regimen for epidural labor analgesia and its combinations need to be individualized for optimal women’s satisfaction [4]. Besides extended epidural anesthesia is recommended as a more effective and safer option for those pregnant women undergoing urgent cesarean delivery by injecting local anesthetic through the epidural catheter, which eliminates the need for spinal or general anesthesia [5, 6].

To establish which combination offers the fastest surgical anesthesia, numerous studies have examined various local anesthetics. It has been shown that 2% lidocaine, epinephrine, bicarbonate, and fentanyl (LEBF) is commonly used for emergency cesarean deliveries and anticipated emergencies with time to prepare, while 3% chloroprocaine (CP) is more commonly used for unanticipated emergencies [6]. CP was reported as the most common and effective local anesthetic for the rapid onset time of anesthesia and limited placental transfer [7, 8]. However, 3% chloroprocaine (CP) required mixing immediately prior to administration and was reported to associate with severe back pain after epidural anesthesia in some literatures [9–11].

One study demonstrated that 0.75% ropivacaine for epidural analgesia has a similar onset time for surgical anesthesia as compared to bupivacaine [12]. Higher concentrations of ropivacaine, such as 0.75%, may allow anesthesia to occur more quickly [12]. Therefore, 0.75% ropivacaine is considered a promising alternative and effective local anesthetic in pregnant women with labor analgesia requiring urgent cesarean delivery. Ropivacaine also has low systemic toxicity [13–15].

Therefore, we conducted this randomized, controlled single-center trial to compare the effectiveness and safety of 0.75% ropivacaine for extended epidural anesthesia with 3% chloroprocaine in pregnant women with labor analgesia requiring urgent cesarean delivery.

## Methods

### Study design

This was a single-center, double-blind, randomized, controlled study that was conducted from November 1, 2022, to April 30, 2023, and approved by The Ethics Committee of Hangzhou Women’s Hospital on September 15, 2022 (IRB: 2022-K(9)-07). Forty-five pregnant women who underwent urgent cesarean delivery while under labor analgesia were selected and randomized in a 1:1 ratio to the 0.75% ropivacaine (RP group) or 3% chloroprocaine (CP group) after providing written informed consent. Inclusion criteria were: (1) being 18 years or older; (2) having an ASA physical status of 1 or 2; (3) being over 36 weeks of gestation; (4) having a singleton pregnancy; (5) having effective labor analgesia (defined as requiring two or fewer intrapartum epidural supplements of 0.2% ropivacaine); and (6) requiring urgent cesarean delivery (category 2 or 3).

Exclusion criteria were: (1) receiving epidural supplementation less than two hours before cesarean delivery or intramuscular pethidine within four hours; (2) requiring emergency cesarean delivery (category 1); (3) having severe fetal anomalies; (4) having pre-eclampsia⁄eclampsia, antepartum hemorrhage, or any form of cardiac disease; (5) being shorter than 152 cm or weighing more than 115 kg; (6) having an allergy to any study drug; or (7) having entered the operating room with a sensory level below T10.

Proposed classification for urgency of caesarean Sect. [16]: (1)emergency: immediate threat to life of woman or fetus; (2)urgent: maternal or fetal compromise which is not immediately life-threatening; (3)scheduled: needing early delivery but no maternal or fetal compromise; (4)elective: at a time to suit the woman and maternity team.

### Randomization and blinding

An independent researcher randomized the postpartum women into the 0.75% ropivacaine group and 3% chloroprocaine group in a 1:1 ratio using numbered sealed envelopes. An independent investigator prepared the injected local anesthetic of 0.75% ropivacaine or 3% chloroprocaine. The independent attending anesthesiologist who administered the local anesthetic drug was not involved in other parts of the study and was unaware of the study drug. An independent anesthesiologist who was not informed of the outcome of the group assignment collected the data. Additionally, the group assignments for the included patients, surgeons, and data analysts were concealed.

### Epidural labor analgesia

Pregnant women were admitted to the operating room where a constant infusion of lactated Ringer’s solution was administered through an intravenous line. All pregnant women underwent epidural block in the L2-L3 position in the right lateral recumbent position. We used a 17-g Tuohy needle to identify the epidural gap by anatomical landmarks and palpation, which was identified by loss of resistantance to the saline technique. After insertion of the epidural catheter, 3 ml of lidocaine 1.5% was injected by the anesthesiologist. If no sign of spinal block was seen after 5 min, 10 ml of local anesthetic solution (0.1% ropivacaine + 2ug/ml fentanyl) was injected for labor analgesia. If a suitable sensory level was not attained within 15 min of the loading dose, further 5-mL aliquots were given every 10 min for the following 20 min, up to a maximum dose of 20 mL. Subsequently, the first dose was administered through a Programmed intermittent epidural anesthetic bolus (PIEB) pump (ZZB-IV; Nanjing Apon Co., Ltd., Nanjing, China) at 60 min after the end of the loading dose. All subsequent PIEB dosing intervals were fixed at 8 ml/h, with Patient-Controlled Epidural Analgesia (PCEA) set at 8 mL/dose, a lockout time of 15 min and a maximum dose of 35 mL per hour, to establish bilateral sensory levels in the T10 block [6]. Subsequently, if analgesia was inadequate, 10 ml of 0.2% ropivacaine was administered.

### Extended epidural anesthesia

If an emergency cesarean section was necessary, the level of pre-existing block to cold sensation was assessed, and motor block was recorded using a modified Bromage score before the pregnant women were admitted to the operating room [17]. The modified Bromage score was as follows: (0) no motor block, able to lift extended leg and flex the knee and ankle; (1) inability to raise extended leg, able to move knees and feet; (2) unable to flex knee; and (3) unable to move lower limb.

An independent attending anesthesiologist administered a test dose of 5 ml of the study drug after confirming negative epidural catheter aspiration and monitored the patient for any indications of an unintentional intrathecal injection. The remaining 15 ml of study drug (0.75% ropivacaine or 3% chlorprocaine) was injected over 3 min. Arterial pressure and heart rate were measured every three minutes.

One investigator marked bilateral T4 levels in the midclavicular line using bilateral nipples as a body surface marker to reduce the previously reported inter-observational differences [18–20]. Cold sensory level was measured at 1-minute intervals with 70% ethanol application. The onset of anesthesia (time from the end of the starting dose to the loss of cold sensation bilaterally to T4 and T5) and the Bromage score to the T4 sensory plane were recorded. The time from the start of anesthesia to the start of surgery was recorded, and patients were instructed to describe any pain using a visual analog scale (VAS), with 0 denoting no pain and 10 denoting the most severe conceivable pain.

An additional 5 mL of study solution was administered if bilateral sensory block was not achieved within 15 min of the epidural extension’s start. If these measures were unsuccessful in providing adequate analgesia, general anesthesia was considered.

Phenylephrine was administered to treat any intraoperative hypotension, defined as systolic blood pressure less than 100 mmHg or greater than a 30% drop from baseline. Toltesetron 5 mg was given intravenously to treat intraoperative vomiting, defined as more than two episodes without hypotension. Intraoperative pain, defined as a VAS score of 3 or higher, was managed with an epidural injection of 5 ml of the study solution (maximum total volume of 25 ml per patient) followed by intravenous esketamine at a dose of 0.25 mg/kg.

### Primary outcome

The primary outcome was the time to onset of T4 block, defined as the time from the start of epidural extension to the loss of cold sensation at the T4 level. If the block did not reach T4 levels within 35 min after the epidural extension began, the onset time was reported as 35 min [6].

Secondary outcomes included the time to onset of T5 block, the modified Bromage score and sensory block at entry into the operating room and at the T4 level, time from induction to the start of surgery (defined as the time from the beginning of anaesthesia to the beginning of surgery), duration of surgery, volume of intraoperative intravenous fluids, vasoconstrictor medication requirements, and intraoperative pain management. Other secondary outcomes were Apgar scores at 1 and 5 min, VAS scores at the beginning of surgery, at delivery, and at the end of surgery, patient satisfaction scores (rated from 0 indicating extreme dissatisfaction to 10 indicating extreme satisfaction), incidence of nausea, vomiting, and shivering, and details of previous epidural analgesia.

### Sample size

There are no existing data in the literature that clearly define a clinically significant reduction in onset time of anesthesia, a difference between the groups to be considered of interest was varied from 5–30% [6, 12, 21]. Based on the study, it takes 12.4 ± 1.1 min for 3% cloprocaine to provide a satisfactory level of anesthesia. A two-sided t-test was conducted with a test power of 95% and α=0.05. Assuming that the time difference between achieving satisfactory anesthesia level with 0.75% ropivacaine and 3% cloprocaine is within 10%, a total of 50 cases or 25 cases per group are required, taking into account a 10% loss to follow-up. G-Power software (version 3.1; Informer Technologies, Inc.) was used for the analysis.

### Statistical analysis

The data were analyzed using SPSS 20.0 (SPSS, Chicago, Illinois, USA) and expressed as mean ± standard deviation and 95% confidence intervals (95% CI). For the primary outcomes, the differences between groups were expressed as median differences (95% CI). Differences between groups were compared using the unpaired t-test, and count data were compared using the X^2^ test or the Fisher exact probability method. Continuous variables with non-normal distribution were analyzed using nonparametric tests (Mann-Whitney U test). VAS scores were analyzed using generalized estimation equations. A two-sided p-value of < 0.05 indicates statistical significance.

## Results

**Fig. 1:**
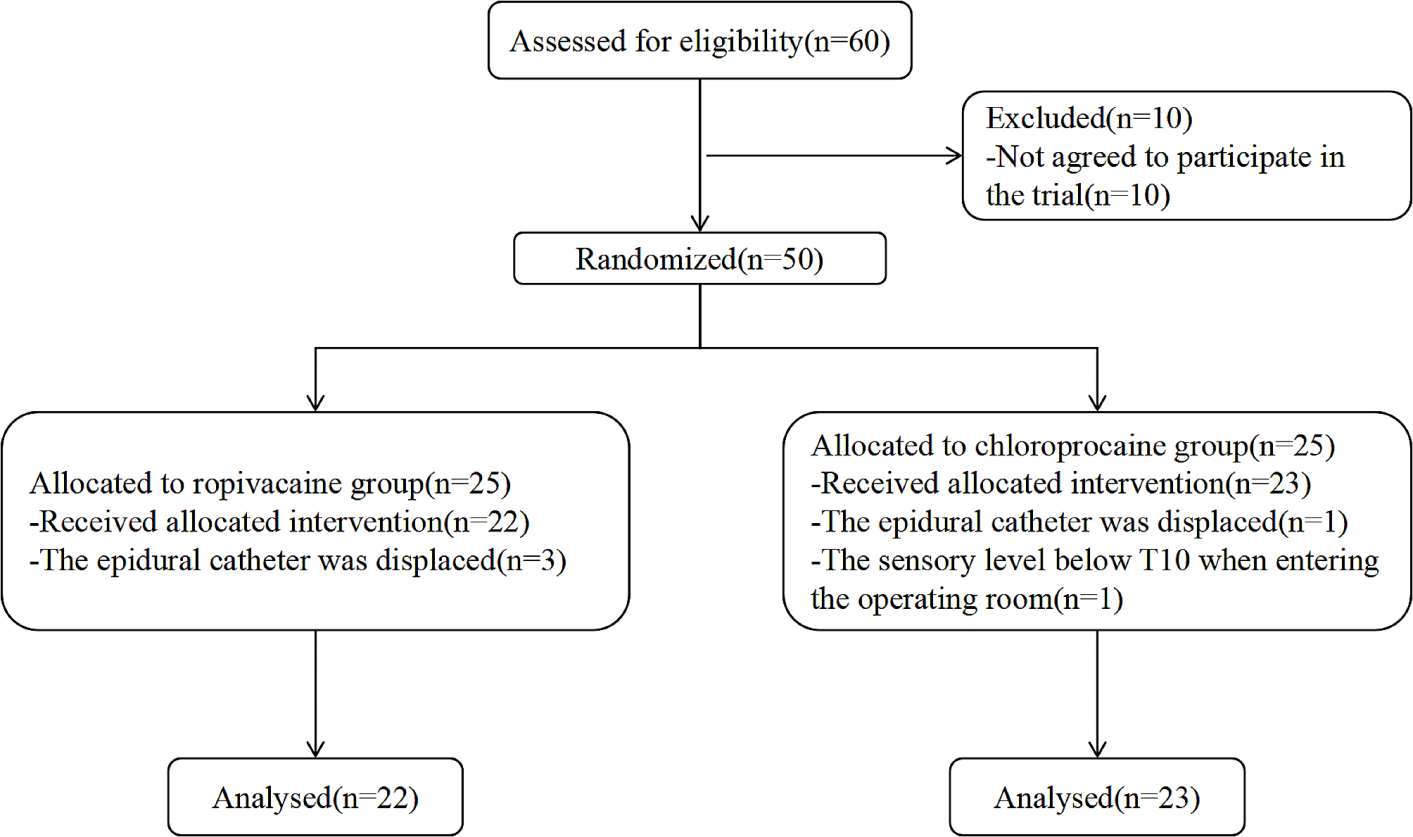
Consolidated Standards of Reporting Trials statement flow diagram

A total of fifty postpartum women agreed to participate in the trial, of whom twenty-two from the RP group and twenty-three from the CP group completed the study protocol. Three and one pregnant women were excluded from the protocol for epidural catheters were displaced before the top-up was given in the RP and CP group respectively. One pregnant woman in the CP group entered the operating room with a sensory level below T10. There were no other deviations from the study protocol (Fig. 1). There were no significant differences in demographic data and indications for cesarean section between the two groups (Table 1).

**Table 1:** Characteristics of patients receiving epidural ropivacaine 0.75% or Chloroprocaine 3% for emergency caesarean section

	Ropivacaine (*N* = 22)	Chloroprocaine (*N* = 23)	*p*-value
Age(y)	31 ± 3	30 ± 3	0.493
Height(m)	1.59 ± 0.05	1.60 ± 0.04	0.800
Weight(kg)	68 ± 8	70 ± 9	0.584
Body mass index (kg/m^2^)	26.9 ± 2.9	27.3 ± 3.1	0.657
Gestation(wk)	39.6 ± 0.8	39.3 ± 0.6	0.082
Indication for Caesarean section			0.766
Failure to progress	18(81.8%)	18(78.3%)	
Other	4(18.2%)	5(21.7%)	

Values are mean ± SD or number (proportion).

The pre-operative details of extended epidural anesthesia are shown in Table 2, with no statistical differences between the two groups. There were no difference in the duration of analgesia(*p* = 0.768) and total dose(*p* = 0.865) between the two groups. No patient required 0.2% ropivacaine supplementation and no patient required an epidural loading dose of more than 20 mL of 0.1% ropivacaine with 2 ug/mL of fentanyl between the two groups. There was no difference in the time since last top-up(*p* = 0.493), epidural loading dose (*p* = 0.891), preoperative fluids i.v. (*p* = 0.457), sensory block (pinprick) (*p* = 0.317) and modified Bromage score(*p* = 0.215) on entry into the operating room between the two groups (Table 2).

**Table 2:** Details of pre-existing epidural analgesia in patients receiving epidural ropivacaine 0.75% or Chloroprocaine 3% for emergency caesarean section

		Ropivacaine (*N* = 22)	Chloroprocaine (*N* = 23)	*p*-value
Duration of labor analgesia(h)				
Median (Q1, Q3)		7(5,9)	6(4,10)	0.768
Mean ± SD		7 ± 3	7 ± 4	
Total dose (ml)				
Median (Q1, Q3)		77(50,107)	75(57,114)	0.865
Mean ± SD		79 ± 35	81 ± 32	
No.of patients requiring 0.2% ropivacaine		0	0	
Time since last top-up(min)				
Median (Q1, Q3)		36(30,45)	40(30,60)	0.493
Mean ± SD		38 ± 12	41 ± 15	
Epidural loading dose, n (%)				0.891
10 ml		10(45.5)	9(39.1)	
15 ml		9(40.9)	11(47.8)	
20 ml		3(13.6)	3(13)	
Sensory block (pinprick) on entry into the operating room, n (%)				0.317
T6		1(4.5)	4(17.4)	
T8		10(45.5)	11(47.8)	
T10		11(50)	8(34.8)	
Modified Bromage score* on entry into the operating room, n (%)				
0		22(100)	20(87)	0.215
1		0(0)	1(4.3)	
> 2		0(0)	2(8.7)	
Pre-operative i.v. fluids(ml)				
Median (Q1, Q3)		900(500,1200)	700(500,1000)	0.457
Mean ± SD		961 ± 589	800 ± 392	

Values are mean ± SD, median (interquartile range) [range], or number (proportion).*0 = able to lift straight leg; 1 = able to bend knee and ankle; 2 = only able to bend ankle; 3 = unable to move leg.

**Fig. 2:**
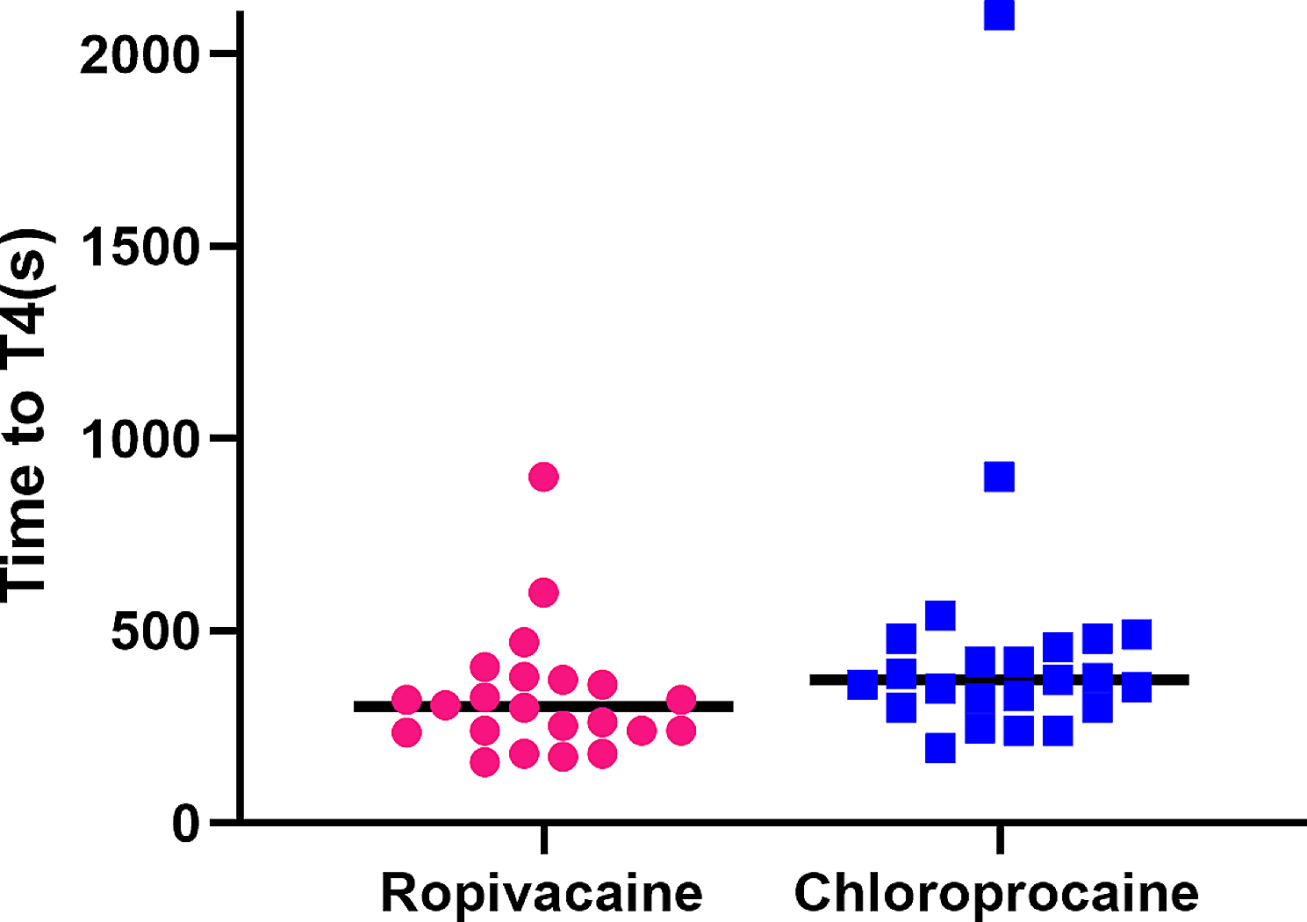
Time to loss of cold sensation to T4 after 20 ml epidural ropivacaine 0.75% or chloroprocaine 3% for 3 min, used to extend low-dose epidural analgesia for emergency Caesarean section. Horizontal lines indicate medians

The RP group tending to have a shorter in the time to loss of cold sensation in the T4 with a median difference of 69 s as compared to the CP group (303, 95%CI 255 to 402 S vs. 372 ,95%CI 297 to 630 S, *p* = 0.024, Table 3; Fig. 2).

**Table 3:** Intraoperative Data and patient satisfaction

		Ropivacaine (*N* = 22)	Chloroprocaine (*N* = 23)	*p*-value
Onset time to T4 block(s)				
Median (Q1, Q3)		303(239,375)	372(300,480)	0.024
Mean ± SD		328 ± 165	464 ± 384	
Onset time to T5 block(s)				
Median (Q1, Q3)		245(201,300)	255(240,420)	0.165
Mean ± SD		249 ± 81	323 ± 167	
Modified Bromage score* on T4, n (%)				0.185
0		0(0)	3(13)	
1		6(27.3)	4(17.4)	
> 2		16(72.7)	16(69.6)	
Induction to surgery start (min)				
Median (Q1, Q3)		12(9,14)	11(10,14)	0.575
Mean ± SD		12.0 ± 2.8	11.7 ± 3.7	
Intraoperative pain treatment, n (%)				
Esketamine		0(0)	0(0)	not applicable
Epidural LA		1(4.5)	8(34.8)	0.011
Patient satisfaction, n (%)				0.040
0–3		0(0)	0(0)	
4–7		0(0)	4(17.4)	
8–10		22(100)	19(82.6)	
Intra-operative i.v. fluids(ml)				
Median (Q1, Q3)		700(600,925)	900(600,1000)	0.154
Mean ± SD		739 ± 186	820 ± 196	
Surgery duration (min)				
Median (Q1, Q3)		48(44,52)	45(43,54)	0.502
Mean ± SD		48 ± 6	48 ± 9	

Values are mean ± SD, median (interquartile range) [range], or number (proportion). *0 = able to straight-leg raise; 1 = able to flflex knees and ankles; 2 = able to flex ankles only; 3 = unable to move legs.

**Fig. 3:**
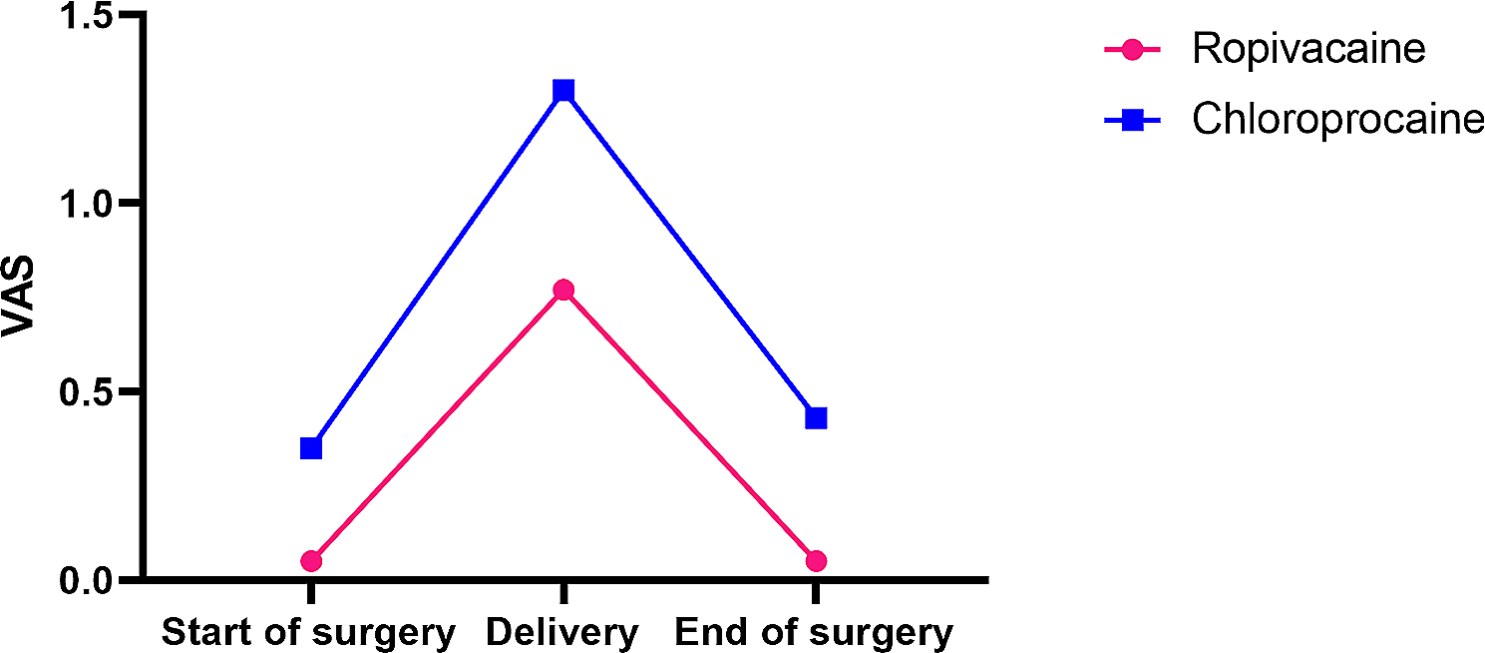
Pain assesment by visual analog scale

There was no statistical difference between the two groups in the time to loss of cold sensation in the T5 ( *p* = 0.165) and in the degree of motor blockade(*p* = 0.185) (Table 3). There was no difference in the time of induction to surgery start between the RP and CP groups (*p* = 0.575). Lower intraoperative VAS scores (*p* = 0.023), less additional local anesthetics requirement (4.5% Vs 34.8%, *p* = 0.011) and more pregnant women give 8–10 points of satisfaction (100% vs. 82.6%, *p* = 0.040) in the RP group than that in the CP group (Table 3; Fig. 3).

No significant difference was found in intraoperative hypotension (9.1% Vs. 26.1%, *p* = 0.136), intraoperative complications, Apgar scores at 1 and 5 min (*p* = 0.323) between the two groups. Two and four pregnant women experienced perioperative nausea (9.1% VS. 17.4%), one pregnant woman experienced vomiting in each group, six and seven pregnant women (27.3% VS. 30.4%) developed shivering in the RP and CP group respectively. In the CP group, one pregnant woman developed severe respiratory distress because of blocks up to T1 (Table 4).

**Table 4:** Neonatal outcomes and Side effects

	Ropivacaine (*N* = 22)	Chloroprocaine (*N* = 23)	*p*-value
Intraoperative hypotension, n (%)	2(9.1)	6(26.1)	0.136
APGAR score at 1 min, n (%)			0.323
< 8	0(0)	1(4.3)	
8–10	22(100)	22(95.7)	
APGAR score at 5 min, n (%)			not applicable
< 8	0(0)	0(0)	
8–10	22(100)	23(100)	
Side effects, n (%)			
Nausea	2(9.1)	4(17.4)	0.413
Vomiting	1(4.5)	1(4.3)	0.974
Shivering	6(27.3)	7(30.4)	>0.999

Values are number (proportion).

## Discussion

In this study, we observed that pregnant women with labor analgesia undergoing urgent cesarean delivery required less intraoperative pain treatment and reported higher satisfaction with 0.75% ropivacaine compared to 3% chloroprocaine. Moreover, 0.75% ropivacaine showed a tendency towards a shorter time to achieve loss of cold sensation and a shorter onset time to T4 block. Therefore, 0.75% ropivacaine could be the preferred drug for extended epidural analgesia during urgent cesarean section in this patient population. In summary, our findings suggest that 0.75% ropivacaine is superior to 3% chloroprocaine for this indication.

During the trial, all pregnant women underwent surgery within 35 min of their top-up, which is consistent with previous studies [6, 12]. The time to achieve loss of cold sensation and the degree of motor block at T4 with 0.75% ropivacaine was equally rapid and reliable as compared to 3% chloroprocaine. The onset time to T4 block was approximately 5–8 min for both ropivacaine and chloroprocaine, and 0.75% ropivacaine had a faster onset time. We only included pregnant women with pre-existing block by labor analgesia, which might shorten the onset time and the difference between 0.75% ropivacaine and 3% chloroprocaine. We also should note that higher concentrations of ropivacaine may allow anesthesia to occur more quickly [12]. Previous studies have shown that the mechanism of local anesthetics action in epidural and spinal anesthesia is more complex [22], and blockage of Na + channels along axons was one of the main mechanisms [23–25]. In our study, the rapid onset of action of 0.75% ropivacaine may be related to the preferential binding of Na + channels, since ropivacaine was used during the epidural labor analgesia. The full effect of local anesthetics is likely to involve other sites [23–25], future research is still needed to clarify this concept.

Chloroprocaine demonstrated a rapid and reliable onset of action time, which is consistent with previous studies [6]. We also documented the onset time to reach T5, and the onset time of 255 s (median) for chloroprocaine was faster than previous studies (480 s) [21], it might due to only pregnant women with of pre-existing block by labor analgesia were included. The most commonly used assessment approach is loss of temperature sensation, and we chose this method because we wanted to apply the same kind of testing that clinicians use on a regular basis. Previous studies have shown that a cold sensory level of T5 or higher is required to achieve adequate surgical anesthesia for cesarean delivery [26–28]. Therefore, in our study, the time to achieve a T4 bilateral sensory block level was used as the primary outcome. Although some women in our study did not reach a block level of T4, they all had adequate surgical anesthesia [26].

In this study, only 4.5% of patients required additional intraoperative local anesthetics with the administration of 0.75% ropivacaine, compared to 34.8% of patients with 3% chloroprocaine. The relative short duration of chloroprocaine was the main reason for this difference. For women with epidural catheters, 0.75% ropivacaine is an attractive alternative to 3% chloroprocaine for urgent cesarean delivery as less supplemental analgesia would be needed. In our study, VAS scores showed statistically significant differences between the two groups and were below three throughout the use of 0.75% ropivacaine, which may be related to the long duration of ropivacaine and the provision of a complete blockade [29]. Patient satisfaction ratings were higher in the ropivacaine group, and no patients needed to change to a different neuraxial or general anesthetic.

The decision to extend epidural analgesia for cesarean delivery is made to avoid an excessively high block or systemic toxicity on the one hand, and to minimize delay on the other hand. Safety is an important issue when deciding which drug to use for prolonged epidural analgesia during an urgent cesarean section. Laishley RS et al. showed that a slow (3–5 min) epidural injection of 20 ml of a local anesthetic solution was safe [30, 31]. Price et al. reported blockage of C7 [31], we also observed one pregnant woman with 3% chloroprocaine administration experienced severe respiratory distress due to a T1 block level in this study. This may be related to the fact that when the low pre-existing block level distributes more extensively with this standard dose than high blocks do. Intravascular or intrathecal injections, and subdural catheter placement are extremely improbable if an epidural catheter is used successfully throughout labor [12], furthermore it can usually be ruled out by careful attention to detail.

Previous studies have noted that chloroprocaine can have the advantageous effect of rapid and reliable onset of action when used without any additives [32]. 0.75% ropivacaine does not require pre-preparation, which reduces the risk of bacterial contamination [33]. There is a risk of error in the preparation process, especially if it is done in a hurry [34, 35]. In urgent cesarean sections, the preparation time before the procedure is minimized to reduce the risk to the fetus and mother. The regional anesthetic used in obstetric surgery is known to cause hypotension as a side effect, however 0.75% ropivacaine was able to keep intraoperative blood pressure steady. The advantages of non-invasive hemodynamic monitoring during elective and urgent cesarean deliveries are becoming more and more clear [36]. Studies have shown that Continuous non-invasive hemodynamic monitoring allowed an early detection of maternal hypotension leading to a prompt treatment with satisfactory results considering neonatal well-being [37, 38]. We did not use non-invasive hemodynamic monitoring in our trial. This may provide evidence that 0.75% ropivacaine can keep intraoperative blood pressure steady. There were no significant differences in intraoperative adverse effects between the two groups, which is consistent with previous studies [6].

### Limitations

There are some limitations to the current investigation. First, our study is the small sample size. However, given the absence of any published studies comparing these drugs for urgent cesarean delivery, we believe our results are still valuable. Second, we did not have enough cord gas samples for analysis, and we did not use more comprehensive neurobehavioral tests to assess newborns, as they are relatively insensitive, and the time to onset of action at T4 served as the primary endpoint of our study. Similar to an earlier study [39], the median APGAR score at 1 min and 5 min were 10 respectively, and there was no significant difference between the two groups.

## Conclusions

This study demonstrates that 0.75% ropivacaine is associated with less intraoperative pain treatment, higher patient satisfaction and reduce the onset time compared to 3% chloroprocaine in pregnant women with labor analgesia undergoing urgent cesarean delivery. Therefore, 0.75% ropivacaine may be a suitable drug in pregnant women undergoing urgent cesarean delivery during labor analgesia period.

## Data Availability

The datasets used during the current study available from the corresponding author on reasonable request.

## Abbreviations

ASA: American Standards Association
BMI: Body Mass Index
RP: Ropivacaine
CP: Chloroprocaine
